# OPTIMIZING MEALTIME (OPTIMAL) IN NURSING HOME RESIDENTS WITH ADRD: A PILOT TRIAL OF A PERSON-CENTERED CARE PROGRAM

**DOI:** 10.1093/geroni/igad104.3509

**Published:** 2023-12-21

**Authors:** Wen Liu, Heather Suh, Kristine Williams, Elizabeth Galik, Barbara Resnick, Kathleen Buckwalter, Kimberly VanHaitsma, Barbara Rakel

**Affiliations:** The University of Iowa, Iowa City, Iowa, United States; The University of Iowa, Iowa City, Iowa, United States; University of Kansas, Lawrence, Kansas, United States; University of Maryland School of Nursing, Baltimore, Maryland, United States; University of Maryland, College Park, Maryland, United States; The University of Iowa, Iowa City, Iowa, United States; University of Maryland, College Park, Maryland, United States; The University of Iowa, Iowa City, Iowa, United States

## Abstract

Nursing home (NH) residents living with dementia (residents) commonly experience behavioral symptoms during mealtime that negatively impact nutrition and function. Residents may not receive optimal, person-centered mealtime care (PCMC) due to multilevel factors. PCMC training that teaches staff to actively engage residents in eating is a prioritized modifiable factor. We developed Optimizing Mealtime (OPTIMAL), a multifaceted, evidence-based, theoretically grounded PCMC program with five components: community stakeholder engagement, environment/policy assessment/modifications, staff education, individualized care planning, and ongoing motivation. OPTIMAL is designed to create and maintain a supportive PCMC culture/environment through appropriate assessments and modifications of factors to optimize mealtime care and outcomes. Our pilot trial (single-group repeated measures design, stakeholders’ interviews) demonstrated OPTIMAL was feasible, acceptable, and perceived useful as evidenced by high recruitment (74%, 77%) and retention (94%, 99%) rates for residents and staff, successful completion of data collection/coding and intervention delivery, and positive stakeholders’ feedback. We recruited 95 staff (age=45±12.1 years, 8.5% male, 12.3% non-white, 89% experienced mealtime care challenges) and17 residents (age=74±7.9 years, 88.2% male, 11.8% non-white) from 8 memory care units of one NH. Stakeholders perceived OPTIMAL was useful for staff who provide care to residents with and without dementia who need assistance and/or experience behavioral symptoms during mealtime. Videotaped observations of dyadic mealtime interactions (N=265) at baseline and 6- and 12-weeks following OPTIMAL showed that resident functional impairments and resistive behaviors during mealtime decreased during the study period. Findings provide preliminary evidence for feasibility, acceptability, and usefulness of OPTIMAL in NH staff and residents with dementia.

